# Prostate-specific antigen density values among patients with symptomatic prostatic enlargement in Nigeria

**DOI:** 10.1186/s12957-016-0921-6

**Published:** 2016-06-29

**Authors:** Emeka I. Udeh, Ikenna I. Nnabugwu, Francis O. Ozoemena, Fred O. Ugwumba, Adesina S. O. Aderibigbe, Samuel R. Ohayi, Kevin N. Echetabu

**Affiliations:** Department of Surgery, University of Nigeria, Enugu, Nigeria; Department of Pathology, ESUT University Teaching Hospital, Park Lane, Enugu, Nigeria; Department of Radiology, University of Nigeria Teaching Hospital, Enugu, Nigeria; Department of Surgery, University of Nigeria Teaching Hospital, Enugu, Nigeria; Department of Surgery, Faculty of medicine, College of medicine, University of Nigeria, Enugu, Nigeria

**Keywords:** Prostate-specific antigen density, Nigerian men, Symptomatic prostatic enlargement

## Abstract

**Background:**

This study aims to estimate the prostate-specific antigen density (PSAD) cutoff level for detecting prostate cancer (CAP) in Nigerian men with “grey zone PSA” (4–10 ng/ml) and normal digital rectal examination findings. We addressed this research question: Is the international PSAD cutoff of 0.15 ideal for detecting CAP in our symptomatic patients with “grey zone PSA?”

**Methods:**

Aim: To estimate the prostate-specific antigen density (PSAD) cutoff level for detecting CAP in Nigerian men with “grey zone PSA” (4–10 ng/ml) and normal digital rectal examination findings.

Design: Prospective.

Setting: A tertiary medical center in Enugu, Nigeria.

Participants: Two hundred and fifty-four men with either benign prostatic hyperplasia (BPH) or CAP were recruited.

Intervention: Patients with PSA above 4 ng/ml or abnormal digital rectal examination or hypoechoic lesion in the prostate were biopsied.

Outcome measures: PSAD and histology report of BPH or CAP.

**Results:**

Ninety-seven patients had CAP while 157 had benign prostatic hyperplasia (BPH). Seventy-two patients had their serum PSA value within the range of 4.0 and 10 ng/ml. PSAD cutoff level to detect CAP was 0.04 (sensitivity 95.88 %; specificity 28.7 %).

**Conclusions:**

The PSAD cutoff level generated for Nigerian men in this study is 0.04 which is relatively different from international consensus. This PSAD cutoff level has a positive correlation with histology and could detect patients with CAP who have “grey zone PSA.”

## Background

There is significant morbidity and mortality associated with prostate cancer (CAP), and recent studies have shown that about 64 % of patients diagnosed with CAP die within 2 years in Nigeria [1]. Unfortunately, there appears to be an increase in the incidence of CAP [2, 3]. There are concerted efforts to improve the screening capability of serum total prostate-specific antigen (PSA) necessitating the various modifications in PSA. Prostate-specific antigen density (PSAD) is one of these modifications.

PSAD estimates the PSA secreted per unit volume of prostatic tissue. This is expected to be higher in malignancy. Also, there has been documented variation in PSA secreted per gram of tissue among different races [4]. Asians for instance secrete more PSA per unit gram of tissue compared to Caucasians [4], while blacks secrete even higher PSA per gram tissue than other races when controlled for age, clinical stage, and Gleason grade [5].

Furthermore, the PSAD cutoff level of 0.15 for detecting CAP developed from a study on Caucasians and African Americans [6] has generated a lot of controversies in recent times [7]. Some authorities believe that when applied to other races or environments, it is not very sensitive and as such could miss out malignant prostates if relied upon solely [7].

Unfortunately, in our environment, most patients present to the clinicians only when they are symptomatic. This underscores the need to appraise the role of PSAD cutoff level of 0.15 in the Nigerian setting.

This study undertakes to determine the difference in prostate-specific antigen density values among our patients with symptomatic benign prostatic hyperplasia on one hand and symptomatic carcinoma of the prostate on the other hand and then to estimate the possible PSAD cutoff level with acceptable sensitivity and specificity for detecting CAP among patients with serum total PSA values in the “grey zone PSA” range (>4 to 10 ng/ml) who have benign digital rectal examination.

## Methods

### Study setting

This study took place at the urology clinic of Enugu State University Teaching Hospital Park Lane, Enugu, Nigeria. Enugu is the major commercial city of Enugu state, southeast Nigeria, with a population of 722,664. This was a hospital-based prospective study consisting of patients with symptomatic prostatic disease.

### Study population

This was a hospital-based study and the subjects consisted of patients with BPH and CAP diagnosed for the first time.

### Inclusion and exclusion criterion

The inclusion criteria consisted of all consented patients who had symptomatic prostatic diseases and presented for the first time to the clinic between November 2009 and December 2012 and were subsequently diagnosed to have either benign prostatic hyperplasia (BPH) or CAP. Excluded from the study were patients who have had any form of treatment for BPH or CAP previously. Previous treatments such as 5 alpha reductase inhibitors, gonadotropin-releasing hormone agonist, or orchiectomy do affect PSA levels and prostate volume [8, 9], both of which are used in determining PSAD. Also, excluded were those with coexisting urethral stricture and diabetes mellitus as these could alter bladder dynamics. In addition, those with other causes of lower urinary tract symptoms were excluded.

### Study method

Ethical clearance for the study was obtained from ESUT Teaching Hospital Park Lane Ethical committee.

The sample size was calculated using the statistical formula shown below:

$$ \mathrm{N}\kern0.5em =\kern0.5em \mathrm{Z}2\mathrm{P}\mathrm{Q}\ \left(\mathrm{DEFF}\right)/{\delta}^2 $$

[10]

where *N* = minimum sample size for a cross sectional prospective study design;

*Z* = the standard normal deviation corresponding to 95 % level of significance. The value obtained from the normal distribution table is 1.96. DEFF = estimated design effect = 1;

and *P* = prevalence rate; for BPH, prevalence rate is 21 % = 0.21 [11]; for CAP, prevalence rate is 13.3 % = 0.133 [3].

$$ Q\kern0.5em =\kern0.5em \left(1-P\right) $$

*δ* = absolute precision, i.e., the value required (in percentage points) which in actual term describes the maximum difference between the population rate and the sample rate that can be tolerated; taken for this study to be 10 % (0.01).

$$ N\kern0.5em =\kern0.5em \frac{1.96^2\times 0.21\times 0.79}{0.1^2}\kern0.5em =\kern0.5em 64\kern0.5em \left(\mathrm{B}\mathrm{P}\mathrm{H}\right); $$

$$ N\kern0.5em =\kern0.5em \frac{1.96^2\times 0.13\times 0.867}{0.1^2}\kern0.5em =\kern0.5em 44\kern0.5em \left(\mathrm{f}\mathrm{o}\mathrm{r}\kern0.5em \mathrm{CAP}\right). $$

Since two groups (BPH and CAP patients) were being compared in generating the PSAD cutoff level, the minimum sample size for this study is 64 for each group.

A total of 254 patients who met the inclusion criteria were recruited for the study while 80 patients were excluded. All patients who met the inclusion criteria that presented during the study period were recruited in the study. Diagnosis of prostatic disease in this study was both clinical and pathological. All subjects were evaluated using international prostate symptom score and digital rectal examination. They were screened by serum prostate-specific antigen (ELISA method using-DS-EIA PSA ELISA ITALY via xx Settembre).

The patients subsequently had abdominopelvic ultrasound. Those with serum PSA above 4 ng/ml or abnormal digital rectal examination (DRE) findings or hypoechoic lesion on ultrasound had transrectal prostate biopsy. Ten biopsies were taken using a disposable semi-automatic size 18GTrucut® biopsy needle, five from each prostate side. The specimen of the biopsy were put in a bottle and fixed in 10 % formalin and submitted to pathological department for hematoxylin-eosin staining. The findings were classified as adenocarcinoma or nodular hyperplasia. Histopathological studies were performed by the same pathologist.

The prostate volume was estimated by abdominopelvic ultrasound (a GE logic S expert 052128 model ultrasound) using a 3.5-MHz curvilinear scanner by a consultant radiologist. The PSAD were calculated for all patients by dividing the serum PSA by the prostate volume [12].

Justification for the use of ultrasound in determination of prostate volume was based on findings that have proven that there was no statistical difference in prostate volume measured by transrectal ultrasound compared to abdominopelvic ultrasound [13–16].

Based primarily on the histology results (except for ten subjects with clinical diagnosis of BPH), the subjects were placed into two groups; those with CAP and those with BPH.

The PSAD of the two groups were analysed to generate a PSAD cutoff level for Nigerian men. The PSAD cutoff level was applied to the patients with “grey zone PSA.” These subset of patients had PSA between 4 and 10 ng/ml and also had normal DRE and ultrasound findings. Subsequently, the specificity and sensitivity of the newly generated PSAD was compared with the universally accepted PSAD cutoff level of 0.15 in this “grey zone PSA” group.

### Statistical methods

For statistical analysis, STATA 13 (StataCorp LP, TX, USA) was used to determine correlation and mean of variables. The correlation among variables was determined by Pearson’s correlation coefficient; while linear regression was used to determine relationship between variables. The statistical program GraphPad Prism 5 software (GraphPad Software Inc., CA, USA) was used to demonstrate the best cutoff point for PSAD as well as to calculate its respective sensitivities and specificities to predict CAP. The receiver operating characteristics (ROC) curve was employed to graphically demonstrate the sensitivities and specificities of the PSAD. *P* < 0.05 statistically significant and with a 95 % confidence interval (CI).

## Results

This study took place between November 2009 and December 2012.

A summary of patient characteristics is presented in Table 1. Two hundred and fifty-four patients completed the study. Of the 254 patients, 157 patients had BPH, while 97 had CAP.

**Table 1:** Clinical characteristic of 254 patients who underwent prostate biopsy

	Number	Age (years)	PSA (ng/ml)	Prostate volume (mls)
BPH	157	64.04 ± 14.47	13.71 ± 17.46	93.06 ± 80.72
CAP	97	69.96 ± 11.67	49.86 ± 41.49	94.43 ± 52.11
*T* test		*P* = 0.0021*	*P* = 0.00*	*P* = 0.88

*Significant *p* value

Table 2 shows the characteristics of 72 patients with “grey zone PSA” with normal DRE. There was no statistical difference between the mean prostate volume of patients with CAP and patients with BPH. The mean PSA values between the two groups differ significantly as reflected by the *P* value (0.002).

**Table 2:** Clinical characteristic of 72 patients with “grey zone PSA” values who underwent prostate biopsy

	Number	Age (years)	PSA (ng/ml)	Prostate volume (mls)
BPH patients with intermediate PSA	57	66.25 ± 9.96	5.41 ± 1.77	102.93 ± 87.75
CAP patients with intermediate PSA	15	65.57 ± 20.55	7.06 ± 1.95	96.12 ± 52.42
*T* test		*P* = 0.86	*P* = 0.002*	*P* = 0.78

*P < 0.05 statistically significant

**Fig. 1:**
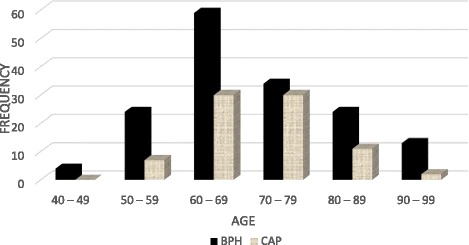
Age distribution of patients in the study

The ages of patients ranged between 40 and 99 years. Forty-six percent of patients were in the age range of 60–69 years (as shown in Fig. 1). Most of the patients with BPH were in the age range of 60–69 years, while for CAP, a greater percentage of patients were in the age range of 70–79 years.

**Fig. 2:**
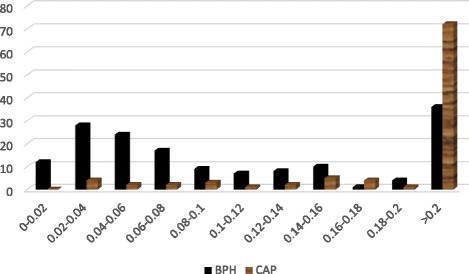
Variations in PSAD between patients with BPH and patients with CAP

Figure 2 shows the variation in PSAD. The BPH patients whose PSAD values were less than 0.08 outnumbered the patients with CAP. However, beyond PSAD value of 0.2 the reverse was the case.

Table 3 shows the mean PSAD value for BPH and CAP which were 0.196 ± 0.325 and 0.77 ± 0.98, respectively. There was statistical difference between mean PSAD values of CAP and BPH.

**Table 3:** Multi-variate analysis for all patients

	Number	Mean PSAD	Median PSAD
BPH	157	0.196 ± 0.325	0.076
CAP	97	0.77 ± 0.98	0.42
*T* test		*P* = 0.00*	

*P < 0.05 statistically significant

Table 4 shows no statistical difference between mean PSAD values of BPH and CAP in the “grey zone PSA.”

**Table 4:** Multi-variate analysis for patients with “grey zone PSA”

	Number	Mean PSAD	Median PSAD
BPH patients with “grey zone PSA” range	57	0.081 ± 0.065	0.070
CAP patients with “grey zone PSA” range	15	0.095 ± 0.053	00.071
*T* test		*P* = 0.45	

*P* < 0.05 statistically significant

The discriminating power to detect CAP as estimated by the ROC curve was 0.8177 for PSAD (area under the curve 0.8188; SD 0.02664; 95 % CI 0.7666–0.8710; *P* = 0.0001).

**Fig. 3:**
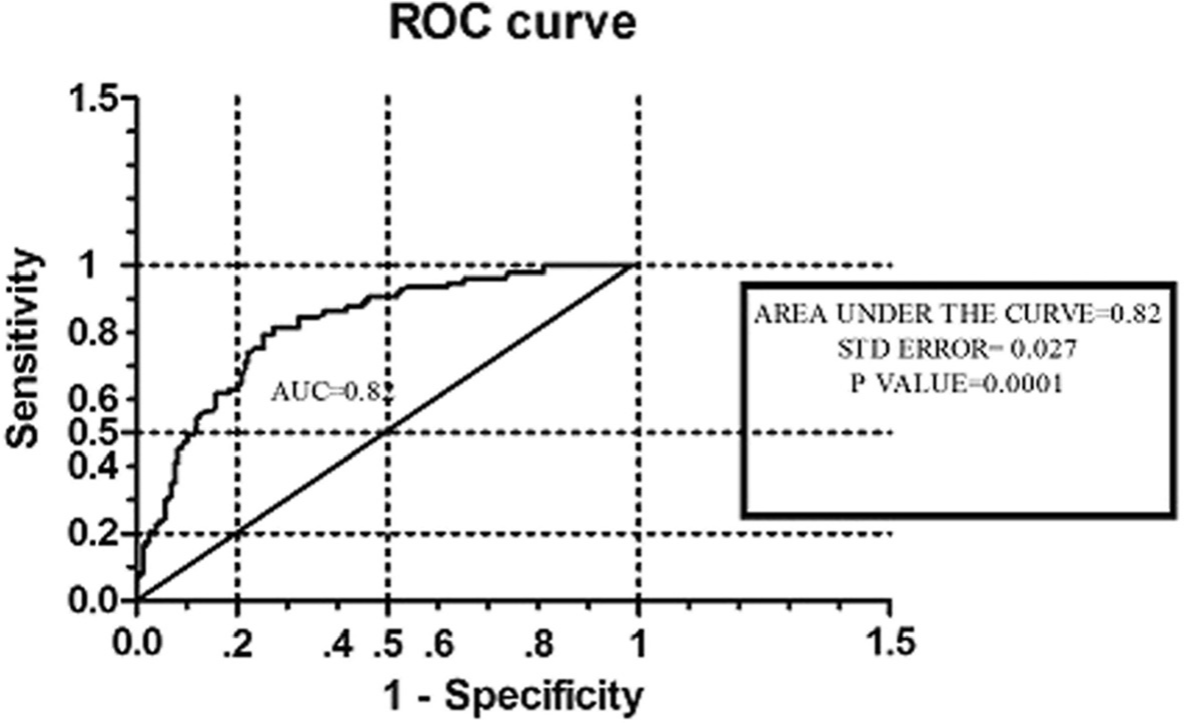
Receiver operating characteristics (ROC) curve depicting diagnostic accuracy of PSA density

Estimates for sensitivity and specificity for different PSAD cutoff points are shown in Fig. 3. The operation characteristics of PSAD at maximum discrimination cutoffs were computed. This was 0.04 for PSAD; the sensitivity was 95.88 % and specificity was 27.8 %.

In establishing the relationship of PSAD cutoff level with histology of patients using Pearson’s correlation coefficient, the correlation coefficient value was 0.31 with a *P* value of 0.00 as shown in Table 5.

**Table 5:** The relationship of PSAD cutoff level (0.04) with histology of patients

	Coefficient *R* value	SD error	*P* value
Pearson correlation coefficient	0.3097	–	0.00*
Linear regression	0.24	0.05	0.00*

*P < 0.05 statistically significant

Table 6 shows the performances of the two different PSAD cutoff levels in detecting CAP in patients with “grey zone PSA.” The sensitivity of the new PSAD cutoff level (0.04) in detecting CAP in the “grey zone PSA” is 86.7 % compared to 33.3 % for the conventional PSAD cutoff level (0.15).

**Table 6:** Performance of the PSAD cutoff levels in screening 72 patients with “grey zone PSA” (4–10 ng/ml)

PSAD cutoff levels	0.15	0.04
Sensitivity	33.3 %: 95 % CI (20–41 %)	86.7 %: 95 % CI (58–98 %)
Specificity	85.7 %: 95 % CI (77–95 %)	20 %: 95 % CI (10–33 %)
Positive predictive value	38.2 %	22.4 %
Negative predictive value	82.8 %	84.9 %
Positive likelihood ratio	2.33: 95 % CI (0.89–6.12)	1.08: 95 % CI (0.85–1.37)
Negative likelihood ratio	0.78: 95 % CI (0.54–1.13)	0.68: 95 % CI (0.17–2.7)

## Discussion

A total of two hundred and fifty-four (254) patients were recruited within the study period. They were all Nigerians from 45 to 99 years of age with mean PSA of 13.71 ± 17.46 and 49.86 ± 41.49 ng/ml for BPH and CAP patients, respectively. Although there was statistical difference in PSA between CAP and BPH, the mean prostate volume was not statistically different between the two groups. This implies that the difference in PSA would not be explained by the volume of the prostate; rather, the distortion in the basement membrane could be the likely explanation. Additionally, the mean prostate volume in our study comparatively was larger than the mean prostate volume recorded in similar studies among Caucasians [17]. However, it did not differ from the findings in a local study carried out by Ugwumba et al. [18] which showed a mean prostate volume of 100.7 mls. Similarly, a study of ultrasonic determination of prostate volume in Nigerian men with symptomatic BPH done by Badmus et al. [19] had revealed a mean prostate volume of 83.79 mls. Likewise, another study on peri-operative blood transfusion in open suprapubic transvesical prostatectomy: relationship with prostate volume and serum total prostate-specific antigen revealed a mean prostate volume of 90.4 cm^3^ for the Nigerian population [20]. These findings may suggest that our study population presented with significantly large prostatic volumes.

For PSAD levels below 0.08, patients with BPH appear to be more in number; beyond 0.2, those with CAP predominated. The operation characteristics of PSAD at maximum discrimination cutoffs were computed as 0.04 with sensitivity of 95.88 % and specificity of 27.8 %. The PSAD cutoff level of 0.04 was strongly positively correlated to the histology of subjects. The new PSAD cutoff level of 0.04 is more sensitive than the previously accepted PSAD cutoff level of 0.15 for detecting CAP when applied to patients with “grey zone PSA” who are symptomatic.

The observed variation in PSAD between BPH and CAP noted in this study seems to agree with earlier established facts that cancer of the prostate tend to produce more serum PSA than BPH. It is known that although benign prostatic tissue secretes more PSA per gram tissue, PSA is confined within the organ because of intact blood basement membrane barrier [21]. Conversely, though carcinoma of the prostate secretes less PSA per gram tissue compared to BPH, due to distorted blood basement membrane barrier, a greater portion of PSA is released in to the blood stream including the complex forms [22].

The ideal cutoff level of PSAD for detecting CAP has remained contentious in recent times. The previously adopted cutoff level of 0.15 has come under vivacious criticism from many scholars. Lujan et al. [7] in their study, in which they dismissed the idea of using PSAD cutoff level of 0.15 for detecting CAP, reported that multivariate analysis failed to demonstrate any significant association between PSAD (based on cutoff level of 0.15) and biopsy results. Moreover, if the recommended cutoff of PSAD (>0.15) is used to prompt biopsy (instead of performing biopsies based solely on serum PSA level greater or equal to 4 ng/ml), as much as 30.6 % of the cancers would remain undetected. They proposed that PSAD cutoff level below 0.07 ng/ml/cc (100 % sensitive; 9 % specific) was most relevant in screening within the “grey zone PSA” range. Although, Lujan et al. concluded that PSAD was not relevant in screening patients in the grey zone PSA if the cutoff level of 0.15 was applied. They suggested that if 0.07 ng/ml/cc was applied, it would be more relevant. This agrees with our findings which suggested that a PSAD cutoff level 0.04 ng/ml/cc would be more relevant in screening within the “grey zone PSA” than the recommended cutoff level (>0.15 ng/ml/cc).

This opinion was shared by Benson et al. [23] who in their study conducted on 61 patients reported that only two patients in the subset of CAP had a PSAD of less than 0.05, and none of the patients with BPH had a PSAD greater than 0.117. Based on this, they concluded that a PSAD of greater than 0.15 was abnormal. These studies affirmed that PSAD cutoff level of 0.15 ng/ml/cc will not be relevant for screening. This explains why PSAD was jettisoned as a tool for screening patients with “the grey zone PSA.” However, adopting a PSAD cutoff level of 0.04 ng/ml/cc generated a high sensitivity of 95.88 % which made it more appropriate for screening.

Most studies in support of using PSAD to evaluate patients with “grey zone PSA” suggested a PSAD cutoff level of 0.15 [24–26]. One of such recent studies was done by Sfoungaristos et al. [27] in which they estimated an optimal cutoff value of PSA density to be 0.15. This was derived by ROC analysis (area under the curve 0.643, *P* = 0.001, 95 % CI 0.568–0.755). Comparing this with our study, the area under the curve of the ROC (area under the curve 0.8188; SD 0.02664; 95 % CI 0.7666–0.8710; *P* = 0.0001) in our study is different from the generated value in their study, it appeared that selection of PSAD cutoff level in Sfoungaristos et al. [27] study was based mainly on specificity without establishing a convenient tradeoff between sensitivity and specificity. Although, a high specificity will reduce false positive results, thereby reducing unnecessary prostate biopsy; a low sensitivity creates the problem of missing out patients with cancer which is more harmful and damaging to management protocol for CAP. Additionally, it increases the cost of management of the disease and the burden of missing out a patient with CAP far outweighs the advantage of reducing unnecessary prostate biopsy. As such, a balanced tradeoff between sensitivity and specificity must be adopted in deriving a cutoff level for PSAD in order to limit this flaw. In our study, this was put into consideration in deriving the PSAD cutoff level. The performance of the PSAD cutoff level generated in our study, which showed a higher sensitivity than the internationally accepted PSAD cutoff level for patients with “grey zone PSA” (86.7 % and 20 % respectively), attests to the advantage of attaching more weight to sensitivity than specificity in generating PSAD cutoff levels.

The new PSAD cutoff level of 0.04 generated in this study is more appropriate for evaluating patients with symptomatic prostatic enlargement. It may aid the urologist in making decisions for patients with “grey zone PSA.” This may reduce unnecessary prostate biopsy. These findings necessitate a more extensive multi-center study with emphasis on a more balanced tradeoff between sensitivity and specificity in deriving the most appropriate PSAD cutoff level. Perhaps, PSAD may become more relevant in the armamentarium of the urologist in decision-making for cancer patients.

In summary, Nigerian men present with large prostatic volumes compared to Caucasians. It is documented that blacks secrete more PSA per unit tissue than Caucasians [5], implying that large prostate volume may lead to slightly elevated PSA. As such, PSAD estimation will be relevant to our population. Depending on PSAD cutoff level with high sensitivity appears to be relevant for screening unlike PSAD cutoff level with specificity.

### Strengths and limitations of this study

There is a possibility of missed cancers in the grey zone PSA belt as a result of biopsy selection method.

The obvious trade-off of diagnostic testing of reduced specificity as sensitivity is increased would increase the number of patients subjected to unnecessary prostate biopsy.

The sample population is heterogeneous.

## Conclusions

In conclusion, the PSAD cutoff level generated for Nigerian men in this study is 0.04 which is relatively different from international consensus. This PSAD cutoff level has a positive correlation with histology and could detect patients with CAP who have “grey zone PSA.”

## Abbreviations

BPH, benign prostatic hyperplasia; CAP, carcinoma of the prostate; PSAD, prostate-specific antigen density
